# Prognostic impact of history of follicular lymphoma, induction regimen and stem cell transplant in patients with *MYC/BCL2* double hit lymphoma

**DOI:** 10.18632/oncotarget.9473

**Published:** 2016-05-19

**Authors:** Shaoying Li, Annapurna Saksena, Parth Desai, Jie Xu, Zhuang Zuo, Pei Lin, Guilin Tang, C. Cameron Yin, Adam Seegmiller, Jeffrey L. Jorgensen, Roberto N. Miranda, Nishitha M Reddy, Carlos Bueso-Ramos, L. Jeffrey Medeiros

**Affiliations:** ^1^ Department of Hematopathology, The University of Texas MD Anderson Cancer Center, Houston, TX, USA; ^2^ Division of Hematopathology, Vanderbilt University Medical Center, Nashville, TN, USA; ^3^ Division of Hematology/Oncology, Vanderbilt University Medical Center, Nashville, TN, USA

**Keywords:** double hit lymphoma, MYC/8q24, BCL2/t(14;18)(q32;q21), high grade B cell lymphoma

## Abstract

*MYC/BCL2* double hit lymphoma (DHL) has been the subject of many studies; however, no study has systemically compared the clinicopathologic features and prognostic factors between patients with *de novo* disease versus those with a history of follicular lymphoma (FL). In addition, the prognostic importance of several other issues remains controversial in these patients. In this retrospective study, we assess 157 patients with *MYC/BCL2* DHL including 108 patients with *de novo* disease and 49 patients with a history of FL or rarely other types of low-grade B-cell lymphoma. Patients received induction chemotherapy regimens including 61 R-CHOP, 31 R-EPOCH, 29 R-Hyper-CVAD, and 23 other regimens. Thirty-nine patients received a stem cell transplant (SCT) including 31 autologous and 8 allogeneic. Sixty-two patients achieved complete remission (CR) after induction chemotherapy. Median overall survival (OS) was 19 months. Clinicopathologic features were similar between patients with de novo tumors versus those with a history of FL (*P* > 0.05). Using multivariate analysis, achieving CR, undergoing SCT, stage and the International Prognostic Index were independent prognostic factors for OS. Stem cell transplantion was associated with improved OS in patients who failed to achieve CR, but not in patients who achieved CR after induction chemotherapy. In conclusion, patients with *MYC/BCL2* DHL who present with de novo disease and patients with a history of FL have a similarly poor prognosis. Achievement of CR, regardless of the induction chemotherapy regimen used, is the most important independent prognostic factor. Patients who do not achieve CR after induction chemotherapy may benefit from SCT.

## INTRODUCTION

Double hit lymphoma (DHL) is a large B cell lymphoma with concurrent translocations involving *MYC* and another oncogene [1]. Double hit lymphoma represents up to 14% of the patients with aggressive B-cell lymphoma and *MYC/BCL2* DHL is most common, representing approximately 65% of all cases, followed by *MYC/BCL2/BCL6* triple hit lymphoma, ~20%, and *MYC/BCL6* DHL, ~15%. [2–5] *MYC* rearrangement is found in multiple B cell lymphomas including Burkitt lymphoma, diffuse large B cell lymphoma (DLBCL), high-grade B-cell lymphoma not otherwise specified (NOS) (previously known as B cell lymphoma, unclassifiable with features intermediate between DLBCL and Burkitt lymphoma [BCLU]), and rarely other neoplasms [6, 7]. *MYC* is a highly regulated transcription factor that is involved in cell cycle regulation, cell metabolism, mitochondrial biogenesis, nucleic acid synthesis, and apoptosis [8]. When *MYC* is up-regulated due to translocation or other mechanisms, the growth-promoting effects of *MYC* can lead to exuberant cell proliferation, especially in the presence of BCL2 translocation which results in overexpression of the anti-apoptotic protein BCL2, as seen in *MYC/BCL2* DHL.

*MYC/BCL2* DHL has been studied extensively in literature [3, 4, 9–23]. Patients with *MYC/BCL2* DHL usually present with advanced stage disease, extranodal involvement, and high serum lactate dehydrogenase (LDH) levels. Bone marrow (BM) and central nervous system (CNS) involvement are common and the International Prognostic Index (IPI) score is often high-intermediate or high. The morphology of these cases usually resembles that of DLBCL or BCLU. Most cases have a germinal center B-cell immunophenotype and a high proliferation (Ki67) rate. Despite of a variety of treatment approaches, patients with *MYC/BCL2* DHL have a poor prognosis with a median overall survival of less than 2 years [3, 4, 11, 13–15, 17, 19, 23].

Multiple studies on the prognosis of patients with *MYC/BCL2* DHL have reported a number of prognostic factors. Johnson *et al*., in a study of 54 patients, identified high IPI score, BM involvement, BCLU morphology, *IG* partner, and BCL2 expression as predictors of poorer prognosis [11]. In contrast, in a study from MD Anderson Cancer Center (*n* = 129, 93 *MYC/BCL2* DHL), performance status, bone marrow involvement and treatment regimen were independent prognostic factors in multivariate analysis [18]. A multicenter retrospective study of 311 cases of DHL with 270 *MYC/BCL2* DHL showed that WBC count, serum LDH, stage, and CNS involvement are independent prognostic factors in multivariate analysis. Intensive induction was associated with improved progression free survival, but not OS [20]. The results of these studies illustrate the current lack of consensus regarding prognostic factors in patients with *MYC/BCL2* DHL.

Although multiple studies on *MYC/BCL2* DHL have been published, to date no study has systemically compared the clinicopathologic features and prognostic factors between patients with *de novo MYC/BCL2* DHL versus those patients with a history of follicular lymphoma (FL). In addition, the prognostic importance of several other issues in this patient group remains controversial. These questions include the following: 1) Is a history of follicular lymphoma predict a worse prognosis in patients with *MYC/BCL2* DHL? 2) What is the prognostic impact of DLBCL versus high-grade B-cell lymphoma NOS (BCLU) morphology? 3) What is the prognostic impact of the *MYC* translocation partner? 4) Does MYC or BCL2 expression have additional prognostic value; and 5) What appears to be the most effective therapy? To address these issues, we systematically studied the clinicopathologic features of 157 patients with *MYC/BCL2* DHL including 108 with *de novo* disease and 49 patients with a history of low-grade B-cell lymphoma, mostly FL.

## RESULTS

### *De novo MYC/BCL2* DHL

#### Baseline clinicopathologic features

**Figure 1:**
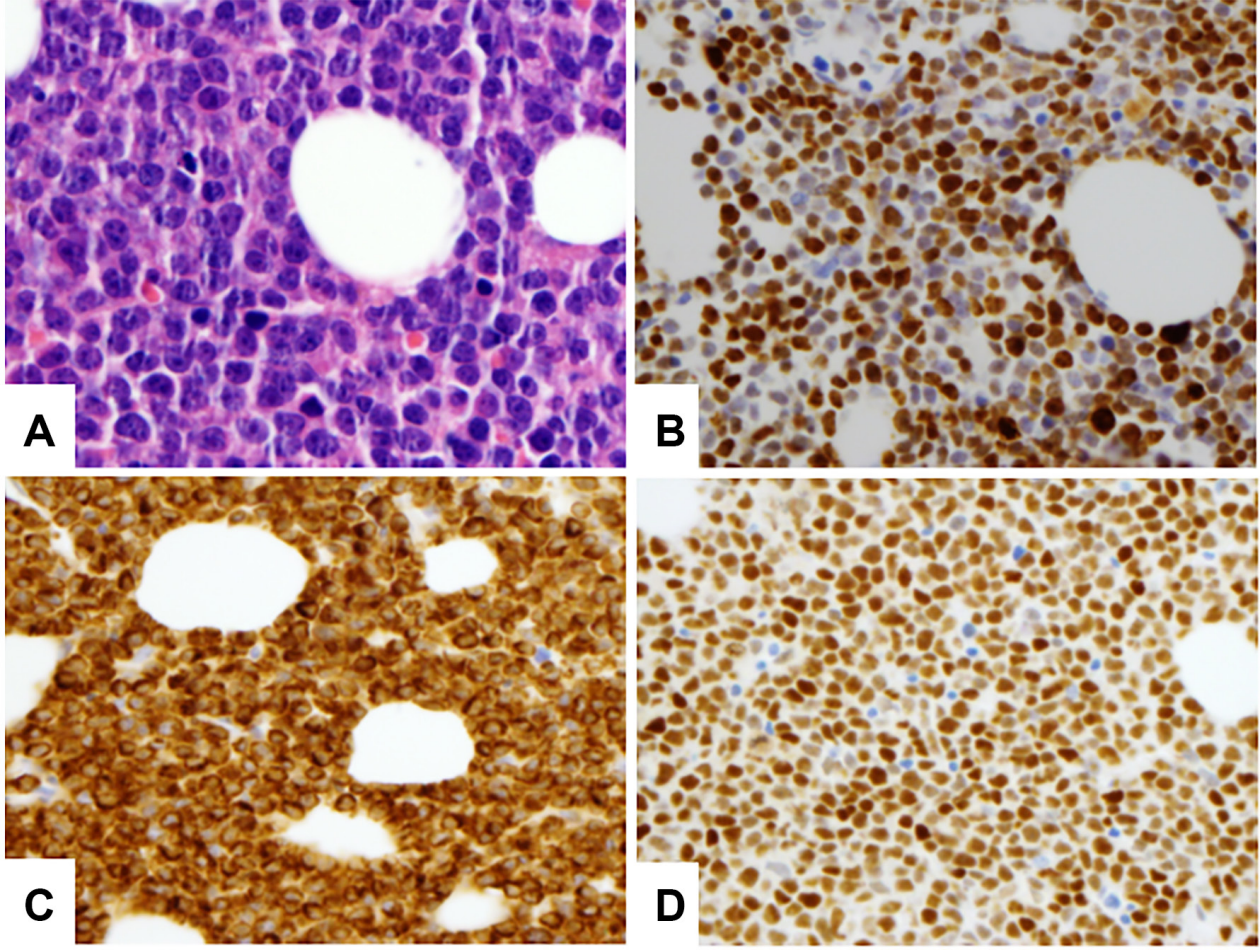
A representative case of DHL with DLBCL morphology (A), high Ki67 proliferation rate (B), BCL2 (C) AND MYC (D) dual expression (A) 600×, B–D. 400×)

There were 108 patients with untreated *de novo MYC/BCL2* DHL, including 73 men and 35 women with a median age of 61 years (range, 18–87). The baseline clinicopathologic features are summarized in Table 1. Half of the patients presented with bone marrow and/or other extranodal sites of involvement by disease. The CNS was involved in 11 of 52 (21%) patients who underwent morphologic evaluation. Most patients presented with an elevated serum LDH, high Ann Arbor stage (III/IV) disease, and high-intermediate to high IPI score. Sixty-four (59%) tumors were classified as DLBCL and 44 (41%) were classified as high-grade large B-cell lymphoma, NOS (previously known as BCLU). Most tumors showed expression of CD10, BCL2, and BCL6 with a high Ki67 proliferation rate. MYC and BCL2 were dually expressed in 72% of tumors assessed (Figure 1; Table 1/column 1). Virtually all tumors had a GCB immunophenotype using the Hans algorithm.[24] In 28 of 30 cases with karyotypic data, the *MYC* translocation partner gene was: 7 immunoglobulin heavy chain (*IGH)*, 16*IG* light chain (*IGK* and *IGL*), and 5 (18%) non-*IG* genes.

**Table 1:** Clinicopathologic features of *de novo*, transformed, and all DHL patients

Features	De Novo DHL (*n* = 108) % (Positive/Evaluated)	Transformed DHL (*n* = 49) % (Positive/Evaluated)	All DHL (*n* = 157) % (Positive/Evaluated)	P (*De novo* vs Transformed)
**Medain Age (yrs, range)**	61 (18–87)	59 (32–86)	61 (18–87)	
**Age ≥ 60 (yrs)**	62 (67/108)	45 (22/49)	57 (89/157)	*0.056*
**Male:Female**	73:35	30:19	103:54	
**Previous FL/B-NHL**		100 (49/49)	31 (49/157)	
**BM positive**	49 (45/91)	51 (23/45)	50 (68/136)	1.000
**CNS positive**	21 (11/52)	12 (3/26)	18 (14/78)	0.526
**Extranodal sites ≥ 2**	54 (58/108)	55 (27/49)	54 (85/157)	1.000
**Elevated serum LDH**	83 (71/86)	86 (36/42)	84 (107/128)	0.952
**Stage III or IV**	88 (84/96)	85 (41/48)	87 (125/144)	1.000
**High-Intermediate/High IPI**	82 (75/91)	82 (36/44)	82 (111/135)	1.000
**Morphology**				
**DLBCL**	59 (64/108)	57 (28/49)	58.5 (92/157)	1.000
**BCLU**	41 (44/108)	35 (17/49)	39 (61/157)	0.487
**DLBCL+FL**	0	8 (4/49)	2.5 (4/157)	
**Immunophenotype**				
**CD10**	97 (99/102)	100 (49/49)	98 (148/151)	0.551
**BCL6**	93 (64/69)	87 (26/30)	92 (90/98)	0.448
**BCL2**	92 (87/95)	91 (42/46)	91 (129/141)	1.000
**MYC**	80 (32/40)	80 (16/20)	80 (48/60)	1.000
**MYC/BCL2 Coexpress**	72 (28/39)	70 (14/20)	71 (42/59)	0.810
**Ki67 ≥ 70% (Ki67 range)**	84 (20–100)	84 (20–90)	84 (20–100)	
**GCB subtype**	99 (102/103)	100 (49/49)	99 (151/152)	1.000
**Cytogenetics**				
**Complex karyotype**	100 (30/30)	100 (16/16)	100 (46/46)	1.000
**IGH as MYC partner**	25 (7/28)	50 (6/12)	33 (13/40)	0.130
**Light chain as Partner**	57 (16/28)	25 (3/12)	48 (19/40)	0.150
**Non-IG MYC partner**	18 (5/28)	25 (3/12)	20 (8/40)	0.677
**Initial Chemotherapy**				
**R-CHOP**	40 (40/99)	31 (14/45)	38 (54/144)	0.354
**R-EPOCH**	22 (22/99)	27 (12/45)	24 (34/144)	0.282
**R-HyperCVAD**	23 (28/99)	13 (6/45)	24 (34/144)	0.282
**Other**	9 (9/99)	29 (13/45)	15 (22/144)	**0.005**
**With SCT**	22 (22/99)	38 (17/45)	27 (39/144)	*0.068*
**CR**	48 (48/99)	33 (15/45)	44 (63/144)	0.107
**Median OS (Months)**	18.6	19	19	0.740

#### Treatment and response

Patients with *de novo MYC/BCL2* DHL were treated with different regimens with or without stem cell transplant (SCT). Ninety-nine patients had complete induction regimen and SCT information available. For this group, 40 patients received rituximab, cyclophosphamide, doxorubicin, vincristine, and prednisone (R-CHOP), 22 etoposide, prednisone, vincristine, cyclophosphamide, doxorubicin, and rituximab (R-EPOCH), 28 rituximab, hyperfractionated cyclophosphamide, vincristine, doxorubicin, dexamethasone, cytarabine and methotrexate (R-Hyper-CVAD), and 9 other chemotherapy regimens (including only CHOP in earlier years). After initial induction chemotherapy, 48 patients achieved complete remission (CR), 14 partial remission (PR), and 31 had persistent or progressive disease (primary refractory disease, PRD). Six patients did not complete induction or evaluation. Twenty-two patients also received SCT including 18 autologous and 4 allogeneic; 14 patients were in CR at the time of SCT. The median overall survival was 18.6 months (Table 1).

#### Prognostic factors

**Figure 2:**
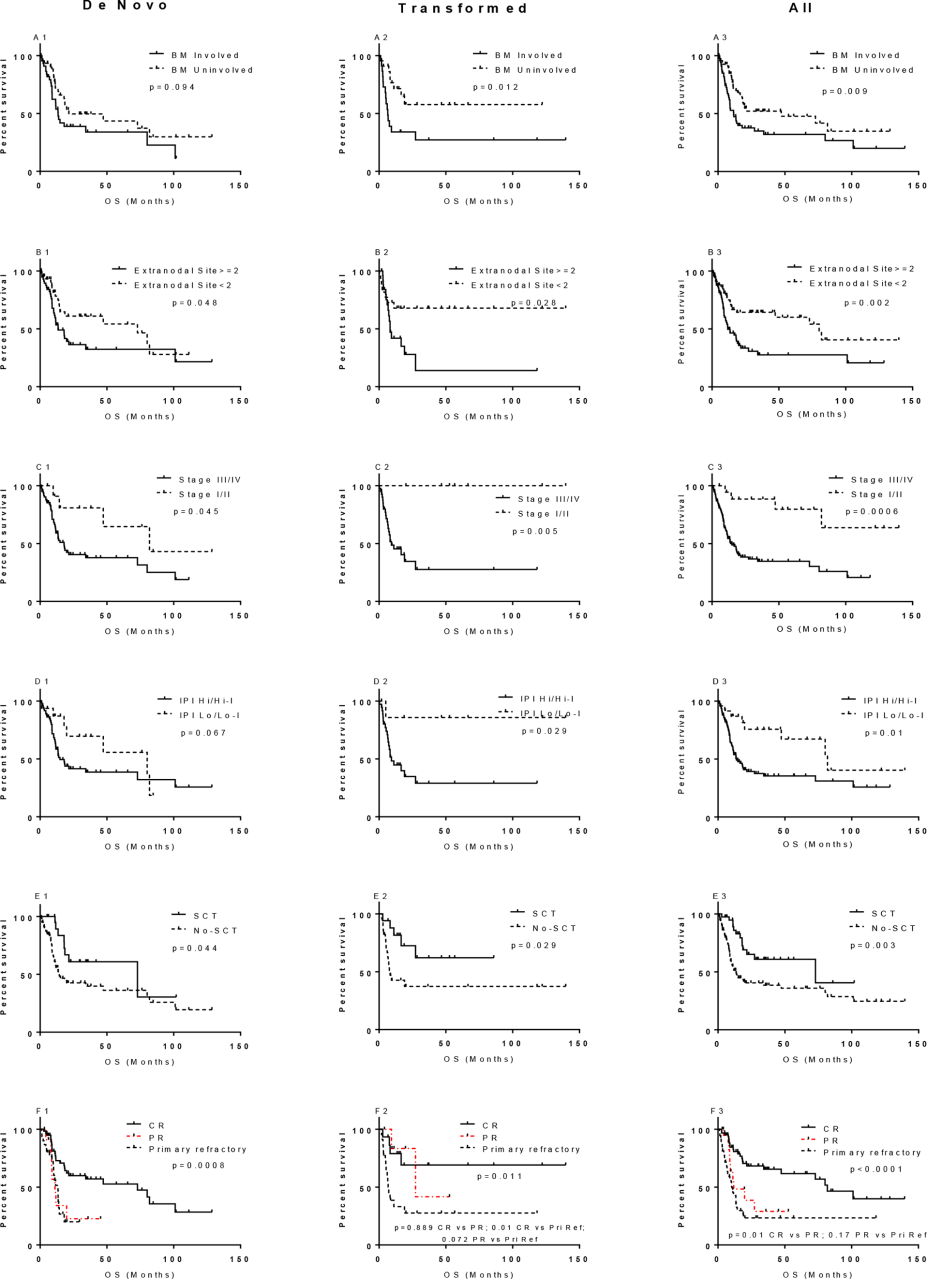
Prognostic significance of bone marrow (BM) involvement, extranodal involvement, stage, IPI, SCT, and CR in overall survival of *MYC/BCL2* lymphoma All label include “1” indicate patients with de novo DHL; those include “2” indicate DHL patients with history of follicular lymphoma; and those include “3” for all DHL.

All 17 collected variables were evaluated by univariate analysis for impact on OS. More than one extranodal site of involvement and high stage (III/IV) were associated with worse OS. Complete response to frontline chemotherapy and SCT were associated with better OS (*p* < 0.05, Figure 2). Bone marrow and CNS involvement (*p* = 0.089) and IPI score tended to be associated with a worse prognosis but had a borderline *p* value (Figure 2, column 1). All other factors assessed, including morphologic classification, BCL2, MYC, or MYC/BCL2 dual expression, *MYC* partner gene, and chemotherapy regimen did not predict prognosis (Supplementary Figure 1, column 1).

### *MYC/BCL2* DHL in patients with history of follicular lymphoma

There were 49 patients with *MYC/BCL2* DHL who had a history of FL (*n* = 46) or other type of low-grade non-Hodgkin B cell lymphoma (*n* = 3). There were 30 men and 19 women with a median age of 59 years (range, 32–86). Patients older than 60 years were less common in this group compared to patients with *de novo* disease (*p* = 0.056). The clinicopathologic features of these patients were similar to those with *de novo MYC/BCL2* DHL (Table 1).

Forty-five patients had complete induction regimen and SCT information available; 14 patients received R-CHOP, 12 R-EPOCH, and 6 R-Hyper-CVAD. Due to the previous treatment for low-grade lymphoma, 13 patients received treatment other than the above mentioned regimens, which were used significantly more often than in patients with *de novo* tumors as expected (29% vs 9%, *p* = 0.005). After induction chemotherapy, 14 patients reached CR, 7 PR, and 22 had PRD. The CR rate was lower for patients in this group compared with patients with *de novo* disease, however, this difference did not reach statistical significance (33% vs 48%, *p* = 0.107; Table 1). Seventeen patients received SCT, 13 autologous and 4 allogeneic, including 10 patients who achieved CR prior to SCT. Relatively more patients received SCT compared to those with *de novo* disease (*p* = 0.068, Table 1).

**Figure 3:**
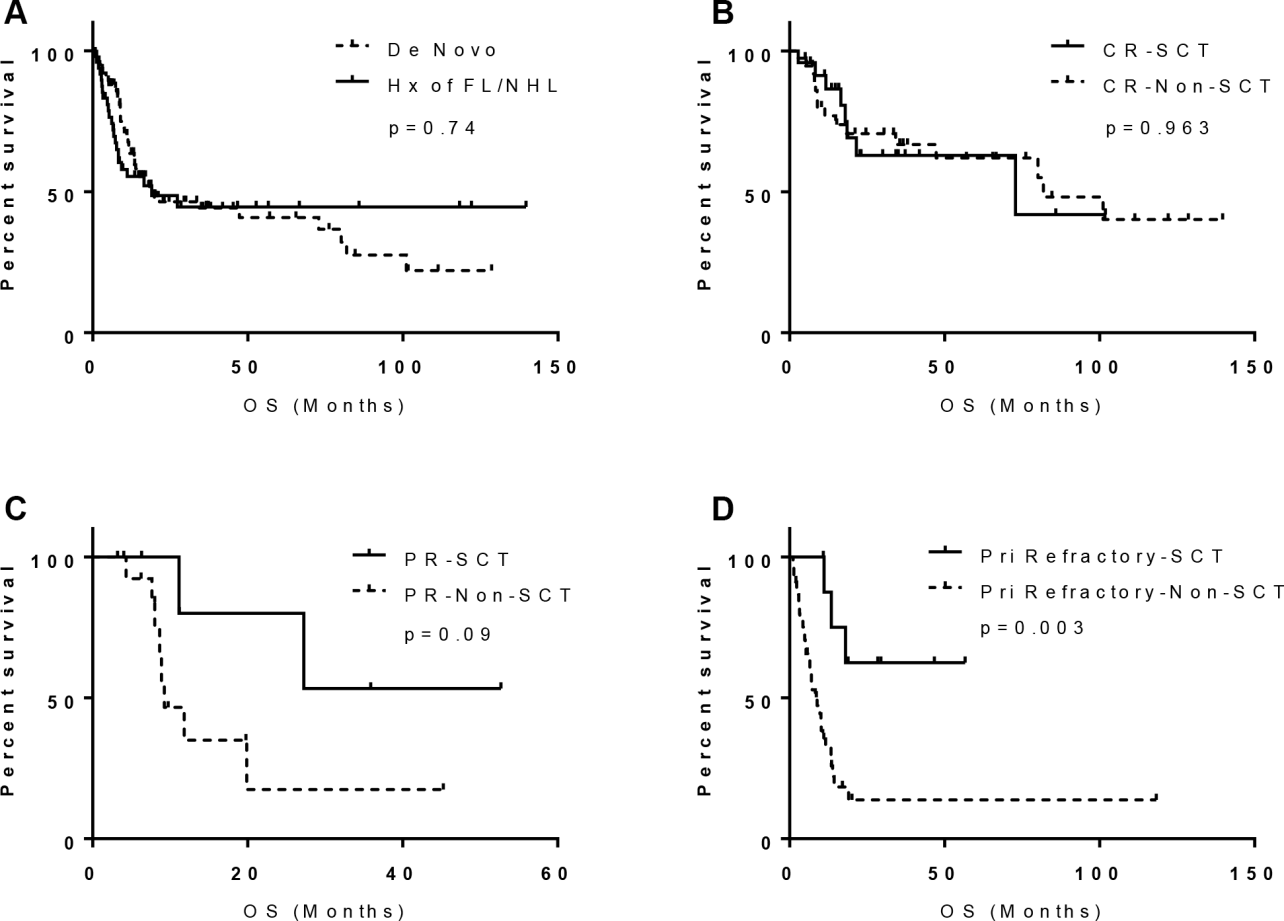
Prognostic significance of history of follicular lymphoma and SCT in *MYC/BCL2* DHL: (A) History of FL; (B) patients achieved CR; (C) patients achieved PR; and (D) patients with primary refractory disease

In a univariate analysis, bone marrow involvement, more than one extranodal site of disease, high Ann Arbor stage (III/IV) and high-intermediate/high IPI were associated with a worse OS (*p* < 0.05), In contrast, achievement of CR and SCT were associated with a better OS (*p* < 0.05, Figure 2, column 2). All other factors assessed including morphologic classification, BCL2, MYC, or MYC/BCL2 dual expression, *MYC* partner gene, and chemotherapy regimen did not predict prognosis (Supplementary Figure 1, column 2). Only 3 patients in this group had CNS involvement and therefore survival analysis was not performed. The median OS of 19 months was similar to that of patients with *de novo* disease, either for all patients (Figure 3A, *p* = 0.74) or only those who did not undergo SCT (Figure not show, *p* = 0.252).

### All *MYC/BCL2* DHL cases

Since the clinicopathologic features and prognosis were similar for patients with *de novo* and for patients with a history of low-grade B-cell lymphoma (46/49 FL), we combined these cases into one group for further assessment of prognostic factors (Table 1, column 3). After a median follow up of 19 months (range, 2 months to 11.7 years), the median OS for all 157 patients was 19 months. The 2-year survival rate was 47%. In univariate analysis, involvement of bone marrow, CNS disease (*p* = 0.033), more than one extranodal site of lymphoma; stage; IPI score; achievement of CR; and undergoing a SCT were prognostic factors for OS (Figure 2, column 3 and Table 2). All other factors assessed including history of FL, morphologic classification, BCL2, MYC, or MYC/BCL2 dual expression, *MYC* partner gene, and type of induction therapy did not predict prognosis (Supplementary Figure 1, Table 2). In patients with bone marrow involvement, we also compared bone marrow involvement by MYC/BCL2 DHL (concordant) versus low-grade B-cell lymphoma (discordant) and there was no significant difference in OS (*p* = 0.24).

**Table 2:** Univariate analysis of clinicopathologic features predictive of overall survival in all DHL patients

Features	Hazard Ratio	95% Confidence Interval	*P*
**Age (> 60 vs ≤ 60 yrs)**	1.20	0.76–1.90	0.440
**Male vs Female**	0.78	0.47–1.27	0.300
**History of FL/B-NHL**	1.22	0.73–2.05	0.440
**BM involvement**	**1.86**	**1.17–3.02**	**0.009**
**CNS involvement**	**1.93**	**1.01–5.03**	**0.049**
**Extranodal sites ≥ 2**	**2.10**	**1.31–3.28**	**0.002**
**Elevated serum LDH**	1.64	0.85–2.81	0.160
**Stage III or IV**	**4.79**	**1.55–4.74**	**0.001**
**IPI (H/H-I vs L/L-I)**	**2.49**	**1.19–3.57**	**0.011**
**BCLU vs DLBCL**	0.88	0.55–1.41	0.601
**BCL2 protein (+ vs −)**	1.65	0.72–3.22	0.270
**MYC protein (+ vs −)**	1.10	0.33–3.64	0.874
**MYC & BCL2 (DE vs Non-DE)**	1.55	0.56–4.06	0.420
**MYC partner (IG vs Non-IG)**	1.54	0.52–4.12	0.474
**Initial Chemotherapy**			
** R-CHOP vs R-EPOCH**	1.27	0.61–2.60	0.545
** R-CHOP vs R-HCVAD**	1.01	0.61–2.00	0.751
**Stem cell transplant**	**0.42**	**0.29–0.78**	**0.003**
**CR after initial chemotherapy**	**0.34**	**0.19–0.50**	**< 0.0001**

Bold: prognostic factors with *P* < 0.05; DE: dual expressor.

The prognostic effect of SCT was further evaluated in patients with CR, PR, or PRD. As shown in Figures 3B–3D, SCT was significantly associated with improved OS in patients with PRD or PR (*p* = 0.003 and 0.09 respectively), but not in patients who achieved CR (*p* = 0.963) after induction chemotherapy. There was no significant statistical difference in OS between patients who received an allogeneic transplant (*n* = 8) compared to those who received an autologous transplant (*n* = 31) (*p* = 0.18), although the patients numbers are small, particularly in the allogeneic SCT group.

All the factors predictive of OS by univariate analysis were entered for multivariate Cox regression analysis (Table 3). After model selection, high stage (III/IV vs I/II) and high IPI (H/H-I vs L/L-I) were negative independent prognostic factors for OS (*P* < 0.05). Achievement of CR and receiving SCT after induction were positive independent prognostic factors for OS (*p* < 0.001). Type of induction chemotherapy, also included in multivariate analysis to exclude the effect of different treatments, was not a prognostic factor for OS (*p* = 0.723 for R-EPOCH vs R-CHOP, and 0.894 for R-Hyper-HCVAD vs R-CHOP).

**Table 3:** Multivariate analysis of clinicopathologic features predictive of overall survival in all DHL patients

Features	Hazard Ratio	95% Confidence of Interval	*P*
**BM involvement**	0.92	0.48–1.80	0.817
**Extranodal sites ≥ 2**	1.12	0.48–2.65	0.793
**Stage III /IV vs I/II**	**34.45**	**4.92**–**241.31**	**< 0.001**
**IPI (H/H-I vs L/L-I)**	**3.00**	**1.14**–**7.77**	**0.026**
**Initial Chemotherapy**			
** R-EPOCH vs R-CHOP**	1.17	0.49–2.84	0.723
** R-HCVAD vs R-CHOP**	0.95	0.47–1.93	0.894
**Stem cell transplant**	**0.22**	**0.10**–**0.46**	**< 0.001**
**CR after initial chemotherapy**	**0.14**	**0.07**–**0.30**	**< 0.001**

Bold: prognostic factors with *P* < 0.05.

## DISCUSSION

*MYC/BCL2* DHL has been well studied in the literature and yet no studies have systemically compared the clinicopathologic features of patients who present with *de novo* disease versus patients with a history of low-grade B-cell lymphoma, mostly FL, and then develop *MYC/BCL2* DHL. Other issues related to prognosis also remain controversial in this patient group which, in part, may be related to the small numbers of patients in many studies as well as the inclusion of different types of DHL.

Others have reported that patients with DLBCL transformed from FL or other type of low-grade B cell lymphoma have a worse prognosis than patients with *de novo* DLBCL, although the difference may be diminished in the era of rituximab therapy [25, 26]. It seems likely that MYC/BCL2 DHL arising in a patient with a history of FL is a manifestation of histologic transformation, but it is unclear if it conveys a poorer prognosis than *de novo* disease [27]. The results in this study show that clinicopathologic features are virtually the same for patients with *de novo* versus transformed *MYC/BCL2* DHL and that OS was similar for both patient groups. These results suggest that a history of FL is not a worse prognostic factor in patients with *MYC/BCL2* DHL, perhaps because the prognosis in both groups is very poor.

The prognostic significance of several clinicopathologic factors in patients with *MYC/BCL2* DHL is controversial in the literature. Pathologic classification of cases of *MYC/BCL2* DHL is one of these issues. One previous study of 54 cases reported that patients with DHL in whom the tumor had DLBCL morphology had a significant better OS than patients with high-grade B cell lymphoma NOS/ BCLU morphology [11]. However, another study of 29 cases found that morphologic classification did not have prognostic implications [28]. The results in this large study suggest that morphologic classification of cases of *MYC/BCL2* DHL does not correlate with prognosis, either in patients with *de novo* disease or in patients with a history of FL.

Another controversial prognostic factor is the translocation partner of *MYC*. Johnson et al reported that cases of *MYC/BCL2* DHL in which *MYC* is partnered with a non *IG* locus had a better prognosis.[11] Two recent studies have shown that the adverse prognostic impact of *MYC* rearrangement correlates with *MYC*-*IG* translocation in *de novo* DLBCL patients treated with immunochemotherapy [29, 30]. In contrast, the study by Aukema and colleagues showed that the *MYC* partner (*IG* versus non-*IG*) had no prognostic significance in *MYC* rearrangement positive B-cell lymphoma (excluding Burkitt lymphoma) [9]. One of the limitations of earlier studies is that the assessed patient cohorts were a mixed group that included *MYC* single hit lymphoma, all types of DHL and triple hit lymphomas, and patients did not receive immunochemotherapy in the study by Aukema *et al*. In this study, there were 40 cases of *MYC/BCL2* DHL in which the partner of *MYC* is known. These 40 cases are a pure group of *MYC/BCL2* DHL, in other words no other DHL or triple hit lymphoma cases, and most patients received immunochemotherapy. Survival analysis showed there was no significant prognostic difference between patients with MYC/BCL2 DHL in which the *MYC* partner was *IG* versus *non-IG*. Additional large and prospective studies focused on *MYC/BCL2* DHL are needed.

In recent years, the use of immunohistochemical analysis has identified a subset of DLBCL cases that are double positive (DPL) or double-expressers of MYC and BCL2. MYC protein expression or MYC and BCL2 dual expression have been shown to be associated with a worse prognosis in patients with DLBCL treated with R-CHOP [31, 32]. However, *MYC/BCL2* DHL and MYC/BCL2 DPL are not concordant; DPL is much more common (20–30% of DLBCL) than DHL (~10% of DLBCL) and not all *MYC/BCL2* DHL are DPL [31–33]. Furthermore, *MYC* translocation is a poor prognostic factor in DPL [33]. Does MYC or MYC and BCL2 dual expression have the same prognostic effect in *MYC/BCL2* DHL as they do in DLBCL? The results in our current study show that both MYC expression alone and MYC/BCL2 double expression have no prognostic significance in patients with *MYC/BCL2* DHL, suggesting that the impact of gene translocation trumps detected protein expression.

The clinicopathologic features in this cohort of patients with *MYC/BCL2* DHL are similar to those reported in the literature. In this study, despite various immunochemotherapy regimens and SCT in a subset of patients, the median OS was 19 months and the 2 year survival rate was 47%. Previous studies have shown conflicting results regarding different chemotherapy regimens and effect of SCT. Some studies showed a possible favorable outcome for patients who received R-EPOCH chemotherapy or SCT [34, 35]. In contrast, other studies demonstrated no prognostic difference for different chemotherapy regimens as well as SCT [11, 14]. Recently, Oki *et al.* [18] reported 129 patients with *MYC* abnormalities including 93 *MYC/BCL2* DHL. In that study, the CR rate was higher in patients who received frontline R-EPOCH (68%) or R-Hyper-CVAD/M (68%) than in patients who received R-CHOP (40%; *P* ≈0.01 for both comparisons). However, only patients receiving R-EPOCH demonstrated a longer EFS (*P* =.004) and OS (*P* =.057) than those who received R-CHOP. In patients who achieved a CR with induction therapy (*n* = 71), the 2-year OS rates were not statistically different between patients with (*n* = 23) or without (*n* = 48) SCT. In another large, retrospective study by Petrich *et al.* that included 311 patients with DHL from 23 academic centers [20], intensive induction regimens (R-EPOCH, R-Hyper-CVAD, and R-CODOX-M) improved PFS (*p* = 0.001) but not OS (*p* = 0.564). Among patients who achieved CR after induction therapy, median OS was similar for those patients who did not undergo SCT (103 months) versus those who underwent consolidation SCT of any type (OS not reached; *P* = 0.14). Both studies suggested that achieving CR with induction therapy, a measure of chemotherapy sensitivity, was a more important predictive factor of outcome than type of induction therapy or received SCT or not.

In this study, patients treated with R-EPOCH showed a trend towards better OS, but the difference between R-EPOCH and R-CHOP was not statistically significant. This result is similar to the study by Petrich *et al.* discussed above, but is different from the results of the study by Oki *et al*. A possible explanation for the discrepancy is that the patient populations were different: our current study group only included the traditional translocation defined *MYC/BCL2* DHL. In contrast, the study from Oki *et al.* included traditional translocation defined *MYC/BCL2* DHL, *MYC/BCL6* DHL, and *MYC/BCL2/BCL6* triple hit lymphoma, as well as cases with extracopies of *MYC, BCL2,* or *BCL6*. In keeping with other studies, the results we present also show that achieving CR is an independent prognostic factor for OS regardless of type of frontline chemotherapy. Although SCT was an independent prognostic factor for OS in this patient cohort, further analysis showed that SCT did not play a role in prognosis if patients reached CR. However, patients who failed to achieve CR had a significant better OS when they received SCT compared to those without SCT (*p* < 0.05), an effect that has not been observed previously. Our results suggest that *MYC/BCL2* DHL patients who fail to achieve CR might benefit from SCT. As this study is retrospective, we acknowledge that additional prospective studies are needed to definitively address this issue.

In conclusion, we showed that achievement of CR, use of SCT, stage, and IPI are independent prognostic factors in patients with *MYC/BCL2* DHL and that patients who failed to achieve CR after induction chemotherapy may benefit from SCT. We also show that a history of FL, morphologic classification (DLBCL versus high-grade B-cell lymphoma NOS), MYC expression, BCL2 expression, MYC and BCL2 dual expression, *MYC* translocation partner gene, and induction chemotherapy regimens were not associated with prognosis. To our knowledge, this is the first study that has systemically compared the clinicopathologic features and prognostic factors in patients with *de novo MYC/BCL2* DHL to patients who have a history of FL before developing *MYC/BCL2* DHL.

## MATERIALS AND METHODS

### Case selection

A total of 157 patients diagnosed with *MYC/BCL2* DHL were included in this study. This neoplasm is defined as a large B-cell lymphoma (either DLBCL or high-grade B-cell lymphoma NOS) with concurrent *MYC* and *BCL2* rearrangements. The designation high-grade B-cell lymphoma NOS has replaced the term BCLU in the upcoming new World Health Organization classification [6].

The cases in this study span the years of 2003 through 2015 with most cases accessioned in the recent five years. A small subset of cases has been reported previously [14]. Corresponding medical records were reviewed to obtain clinical information, including a history of lymphoma, number and sites of involvement, Ann Arbor stage, IPI score, treatment regimens, response to therapy, and overall survival. The study has been approved by our institutional review board.

### Immunophenotyping

Imunohistochemical analysis was performed using formalin-fixed, paraffin-embedded tissue sections, either at the time of diagnosis or retrospectively for this study. The panel of monoclonal antibodies used was variable over time but included reagents specific for CD3 and CD20 (Ventana Medical Systems, Tucson, Arizona, USA); CD5, CD10, BCL2, BCL6, and MUM1/IRF4 (Leica Microsystems, Buffalo Grove, IL, USA); MYC (Epitomics, Burlingame, CA, USA); and Ki-67 (MIB- 1) (DAKO, Carpinteria, CA, USA). The cutoffs for positivity for CD10, BCL6, and MUM1 were 30% as were used by Hans and colleagues [24]. The positive cutoffs for MYC and BCL2 were ≥ 40% and ≥ 50% of cells, respectively, as have been used earlier and by others [32, 33, 36–39].

Flow cytometry immunophenotypic analysis was performed using standard multicolor analysis, four-color in cases accessioned earlier and mostly eight-color in recent years. Analysis was performed using FACScanto II or FACS-Calibur cytometer (Becton-Dickinson Biosciences, San Jose, CA, USA) as described previously.[14] Lymphocytes were gated for analysis using CD45 expression and side scatter. The panel was variable and included CD3, CD4, CD5, CD7, CD8, CD10, CD13, CD19, CD20, CD23, CD33, CD34, CD38 and kappa and lambda light chains. All antibodies were obtained from Becton-Dickinson Biosciences.

### Conventional cytogenetic studies and fluorescence *in situ* hybridization

Conventional G band karyotype analysis was performed on 46 MYC/BCL2 DHL cases. The karyotypes were reported according to the 2013 International System for Human Cytogenetic Nomenclature [40].

Fluorescence *in situ* hybridization (FISH) analysis for MYC and BCL2 was performed in all cases using the LSI *MYC* dual-color break-apart and LSI *IGH@BCL2* dual-color, dual fusion probes (Abbott Laboratories, Des Plaines, IL, USA). *BCL6* gene status was tested by FISH using dual color break-apart probe. *MYC/BCL2* DHL cases were identified if they had rearrangements of *MYC* and *BCL2*, but not *BCL6* or 3q27 abnormalities by conventional karyotype. For bone marrow aspirate specimens, FISH was performed by using a freshly dropped slide from a harvested bone marrow or a G-banded slide for metaphase mapping according to the manufacturer's instructions. For formalin-fixed, paraffin-embedded tissue samples, FISH was performed on 4-micron thick tissue sections and fixed onto slides according to the manufacturer's protocol. The signals from 200 nuclei were analyzed.

### Statistical analysis

Overall survival (OS) was calculated from date of diagnosis to the date of death or last follows up. Patient survival was analyzed using the Kaplan-Meier method and compared using the log rank test. Multivariate analysis was performed using Cox proportional hazards model. Statistical analysis was performed using SPSS 23 software. Fisher's exact test was used to compare the clinicopathologic features between patients with *de novo* versus patients with a history of low-grade B-cell lymphoma before *MYC/BCL2* DHL. A *p* value of less than 0.05 was considered significant.

## SUPPLEMENTARY MATERIALS FIGURE


