# Supplementary material for: Prognostic impact of history of follicular lymphoma, induction regimen and stem cell transplant in patients with MYC/BCL2 double hit lymphoma
Source: Oncotarget. 2016 May 19;7(25):38122–32. doi: 10.18632/oncotarget.9473 (PMC5122376; doi:10.18632/oncotarget.9473)
Supplement: Supplementary file 1 [file oncotarget-07-38122-s001.pdf]

Prognostic impact of history of follicular lymphoma, induction regimen and stem cell transplant in patients with *MYC/BCL2* double hit lymphoma

Supplementary Materials

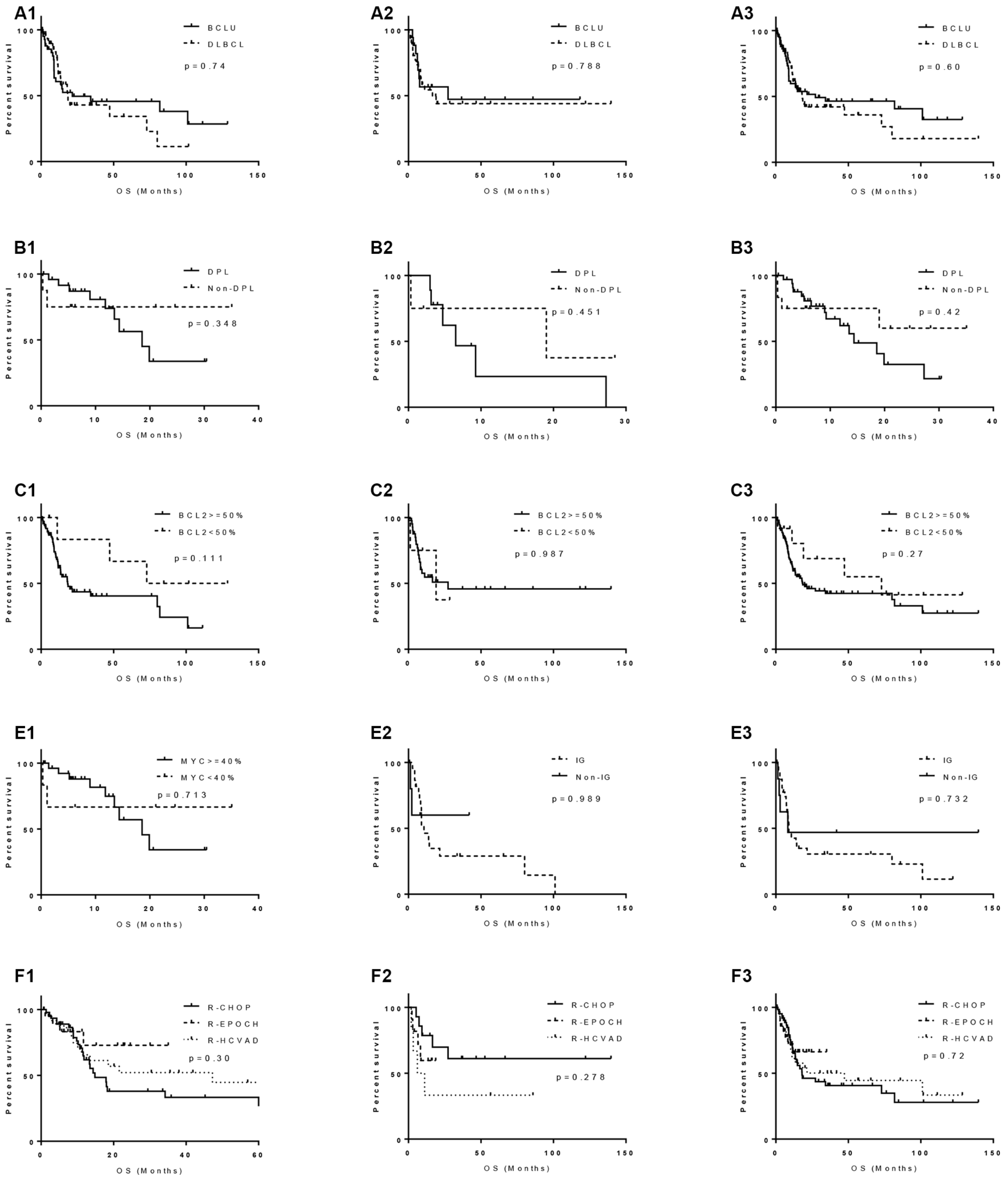

Supplementary Figure S1: Prognostic significance of morphology, MYC & BCL2 dual expression, BCL2 expression, MYC expression, MYC translocation partner, and induction chemotherapy regimen in overall survival of *MYC/BCL2* lymphoma. All labels included "1" indicate patients with de novo DHL; those included "2" indicate DHL patients with history of follicular lymphoma; and those included "3" for all DHL.
